# Investigation of the Photoresponse and Time-Response Characteristics of HDA-BiI_5_-Based Photodetectors

**DOI:** 10.3390/ma15010321

**Published:** 2022-01-03

**Authors:** Yifei Wang, Xiaoping Zou, Jialin Zhu, Chunqian Zhang, Jin Cheng, Junqi Wang, Xiaolan Wang, Xiaotong Li, Keke Song, Baokai Ren, Junming Li

**Affiliations:** 1 Research Center for Sensor Technology, Beijing Key Laboratory for Sensor, Jianxiangqiao Campus, Beijing Information Science and Technology University, Beijing 100101, China; yifeiwang2020@126.com (Y.W.); chunqiancool@163.com (C.Z.); chengjin@bistu.edu.cn (J.C.); 13126706081@163.com (J.W.); wangxl1105@163.com (X.W.); xiaotong252240@163.com (X.L.); renbk2021@163.com (B.R.); li@physik.HU-berlin.de (J.L.); 2 School of Automation, Jianxiangqiao Campus, Beijing Information Science and Technology University, Beijing 100101, China; 3 Beijing Advanced Innovation Center for Materials Genome Engineering, Jianxiangqiao Campus, Beijing Information Science and Technology University, Beijing 100101, China; 4 MOE Key Laboratory for Modern Measurement and Control Technology, Beijing Information Science and Technology University, Beijing 100101, China; 5 Beijing Key Laboratory for Optoelectronic Measurement Technology, Jianxiangqiao Campus, Beijing Information Science and Technology University, Beijing 100101, China; songmengke163@163.com

**Keywords:** photodetector, organic–inorganic hybrid material, thin films

## Abstract

Photoelectric devices can be so widely used in various detection industries that people began to focus on its research. The research of photoelectric sensors with high performance has become an industry goal. In this paper, we prepared photodetectors using organic–inorganic hybrid semiconductor materials with narrow bandgap hexane-1,6-diammonium pentaiodobismuth (HDA-BiI_5_) and investigated the detector photoresponse and time-response characteristics under a single light source. The device exhibits high photoresponsivity and fast response time. The photoresponsivity can reach 1.45 × 10^−3^ A/W and 8.5 × 10^−4^ A/W under laser irradiation at 375 nm and 532 nm wavelengths, and the rise and decay times are 63 ms and 62 ms, 62 ms and 64 ms, respectively. The device has excellent performance and this work can extend the application of organic–inorganic hybrid semiconductor materials in photovoltaic and photodetectors.

## 1. Introduction

A photodetector is a device that converts a light signal into an electrical signal output, and they play a vital role in various industrial and scientific fields such as sensing, communication, and imaging [1,2,3,4,5,6]. With a variety of photodetectors being developed, Ma et al. developed a photodetector based on BaTiO_3_ material, which exhibited an excellent pyroelectric effect, and output current/voltage signals were observed even under slight temperature fluctuations. In addition, the photoresponse at 365 nm was enhanced using the pyrogenic pyroelectric effect, and the corresponding photoconductive gain and responsivity were significantly improved [7]. Chen et al. prepared a PLZT film with large residual polarizability through a low-cost sol–gel method and developed a self-powered UV photodetector with high sensitivity based on it [8]. The narrowest bandgap of organic–inorganic hybrid semiconductor material hexane-1,6-diammonium pentaiodobismuth (HDA-BiI_5_) is 1.89 eV [9]. This material has been used to prepare devices, and D. M. Fabian et al. [10]. prepared solar cells using HDA-BiI_5_ as a light-absorption layer on FTO substrates, only tested the relationship between time and photocurrent using a sunlight simulator, and did not investigate the photodetector photoresponse characteristics. However, we have systematically analyzed the photodetector performance parameters. In addition, Liu et al. prepared conventionally structured solar devices using organic–inorganic hybrid semiconductor material hexane-1,6-diammonium pentaiodobismuth (HDA-BiI_5_) and tested the relevant photovoltaic performance under solar light conditions [11]. Wang et al. then prepared photodetectors without electron transport layers on this basis and found that the high photoresponsivity and specific detectivity made the performance of photodetectors based on HDA-BiI_5_ material greatly improved [12]. The selection of suitable materials and the change of device structure can greatly improve the performance of photodetectors [13,14,15,16,17].

In this paper, we use the organic–inorganic hybrid semiconductor material hexane-1,6-diammonium pentaiodobismuth (HDA-BiI_5_) as the light-absorbing layer to prepare photodetectors with ITO/SnO_2_/HDA-BiI_5_/Spiro-MeOTAD/Au structures and explore the photoresponse and time-response characteristics of the HAD-BiI_5_-based photodetectors. The photoresponsivity of the device can reach 1.45 × 10^−3^ A/W and 8.5 × 10^−3^ A/W under laser irradiation at 375 nm and 532 nm wavelengths, which is more than two-times higher than that of the recently reported literature [12], showing high photoresponsivity and a fast response time. In addition, the effects of different powers on the switching ratio, specific detectivity, and external quantum efficiency are further discussed, which provide a reference for the preparation of photodetectors with organic–inorganic hybrid semiconductor materials in the future, thus further expanding the application of organic–inorganic hybrid semiconductor materials.

## 2. Experimental

Details of the material preparation, characterization, and optoelectronic test platform are available in the previously reported literature [12].

### 2.1. Device Fabrication

#### 2.1.1. Preparation of Electron Transport Layer

SnO_2_ with a purity of 15 wt% was used from Alfa Aesar Chemical Co. (Shanghai, China). The SnO_2_ spin-coating solution was diluted by mixing the purchased 20 wt% aqueous SnO_2_ solution with ultra-pure water (H_2_O) at a volume ratio of 3:8 to form the SnO_2_ spin-coating solution, and the configured SnO_2_ spin-coating solution was ultrasonically cleaned in an ultrasonic vibration cleaner for 5 min. After that, 40 μL of the SnO_2_ solution was spin-coated at 4000 r.p.m. for 30 s, and the procedure was repeated once to ensure that the SnO_2_ spin-coating solution could cover the indium tin oxides (ITO) substrate completely and uniformly. After spin-coating, the substrate is immediately annealed on a hot plate at 150 °C for 30 min.

#### 2.1.2. Preparation of Light-Absorbing Layer

A quantity of 500 mg HDA-BiI_5_ powder was weighed and dissolved in 0.4 mL of N,N-Dimethylformamide (DMF) solution, and the solution was heated and stirred at 90 °C for 4 h until the powder was completely dissolved, at which time the solution turned dark brown. Next, the completely dissolved solution was filtered through a syringe with a 0.22 μm diameter filter tip to remove the residual particles that were not completely dissolved in the solution, and the HDA-BiI_5_ solution for the preparation of the film was obtained. A quantity of 40 μL HDA-BiI_5_ solution was spun at 6000 r.p.m. for 40 s using a pipette. After spin-coating, the samples were immediately annealed on a hot plate at 150 °C for 30 min.

#### 2.1.3. Preparation of Hole Transport Layer

In this experiment, 2,2′,7,7′-Tetrakis[N,N-di(4-methoxyphenyl)amino]-9,9′-spirobifluorene (Spiro-MeOTAD) spin-coating solution was chosen for the preparation of the hole transport layer. In the process of preparing the Spiro-MeOTAD-cavity transport layer, 20 μL of Spiro-MeOTAD spin-coating solution was firstly applied onto the prepared HDA-BiI_5_ light-absorbing layer film with a pipette, and then spin-coated on a homogenizer at 3000 r.p.m. for 30 s. Oxidation was carried out in a drying urn overnight, and the cavity transport layer was prepared after the oxidation.

#### 2.1.4. Preparation of Electrodes

In order to prepare a vertically structured photoelectric device, a gap of about 3 mm should be left at the edge of the conductive side of the ITO substrate before preparing the counter electrode, and the material prepared above should be cleaned up, so that this part of the electrode can be prepared on the ITO conductive glass substrate to form a vertically structured device. In this experiment, Au was chosen as the metal counter electrode and prepared using a vacuum vapor deposition apparatus.

## 3. Results and Discussion

**Figure 1:**
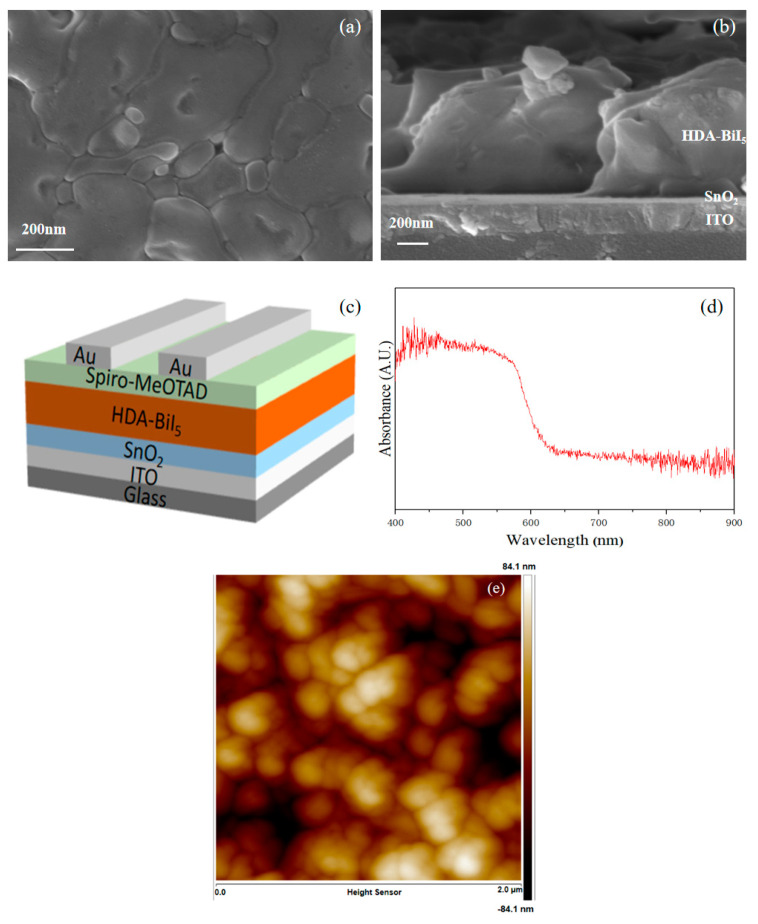
SEM images of HDA-BiI_5_ layer: (**a**) top view; (**b**) cross-sectional view; (**c**) Schematic diagram of the device structure; (**d**) UV-vis spectrum; (**e**) AFM topography.

After the preparation of HDA-BiI_5_ film and its characterization—for XRD data see the previous article [12]. Through Figure 1a,b,e, we can see that the grain boundaries are obvious, the grains are extruded together with each other, the pinholes are less but the surface is not too flat, and overall, the thin HDA-BiI_5_ film is of good quality, the film thickness is about 750 nm, and the good film quality can reduce the occurrences of short-circuiting and enhance the light absorption ability, thus improving the performance of the device [11,18,19]. Then, a layer of Spiro-MeOTAD is spin-coated on the light-absorbing layer to prepare the device structure as in Figure 1c. In addition, from Figure 1d it can be clearly seen that the wavelength of the absorbed light is less than 660 nm and the absorption edge is about 640 nm, indicating a bandgap of about 1.93 eV, which corresponds to the previously reported literature [12].

**Figure 2:**
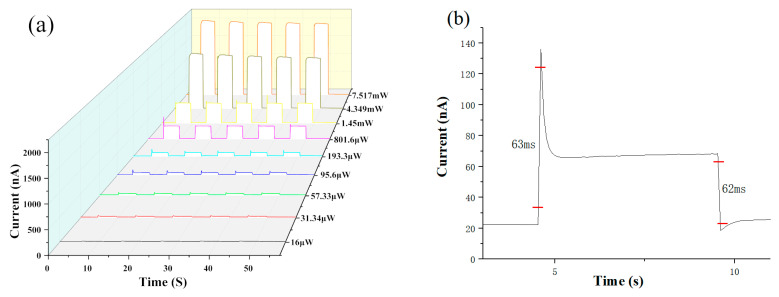

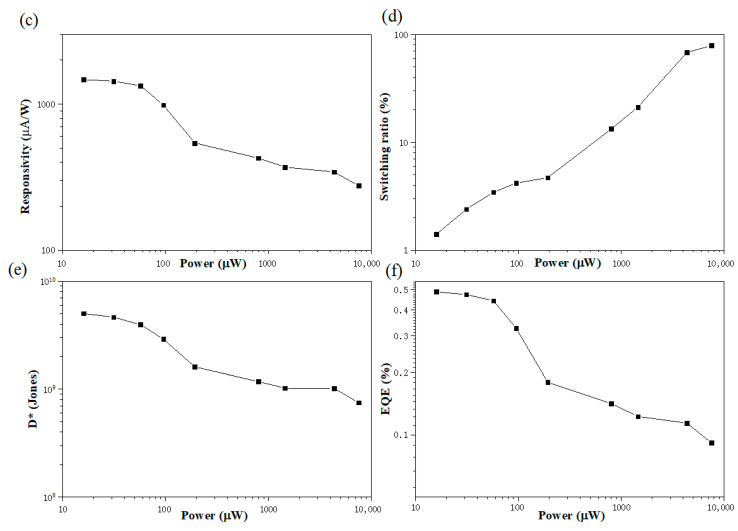
The time-response and photoresponse characteristics of HAD-BiI_5_-based photodetectors under a zero-bias 375 nm laser: (**a**) photocurrent versus time plots of device; (**b**) response time of HDA-BiI_5_-based photodetector (375 nm, 95.6 μW); (**c**) photoresponsivity; (**d**) switching ratio; (**e**) specific detectivity; (**f**) external quantum efficiency.

Figure 2a shows photocurrent versus time plots of the device under a zero-bias 375 nm laser with a switching time of 5 s. It can be seen that when irradiated by a 375 nm laser, this device can rapidly generate a repeatable photocurrent with a maximum of 2100 nA, which is due to the rapid increase of carriers in a short amount of time as the light power increases. At the same light power, the photocurrent at each light-on maintains a stable value and drops rapidly to the original value after the light-off, which means that this device has reached the maximum photocurrent within 5 s. The cycle dependence of the photoresponse to the light switch shows good stability and repeatability, and the device responds quickly at different powers. We performed five consecutive switching operations and found that the photocurrent changes are small for each of the five times. As seen in Figure 2b, the device has a very low dark-current of about 20 nA, and the very low dark-current is a guarantee of the reliability of the device. In addition, the response time is one of the most important parameters that determine the performance of the detector. The rise time of our photodetector is about 63 ms and the decay time is about 62 ms, which shows that the device can quickly generate the photoresponse and reflects the stability of the device under frequent switching.

From Figure 2c–f, it can be seen that the photoresponsivity, specific detectivity, and EQE gradually decrease with the enhancement of optical power. At low power, the photoresponsivity can reach up to 1.45 × 10^−3^ A/W, and the specific detectivity is about 5.0 × 10^9^. As can be seen from Table 1 [7,8,10,11,12,20,21,22,23,24,25], the photoresponsivity is more than twice that of other devices with similar structural materials. The high photoresponsivity is due to the integrity of the device structure. The electron-hole pairs in the light-absorption layer separation are produced under the excitation of light energy, the modification of the electron transport layer is increased, and a large number of electrons can pass through the electron transport layer more easily for better transport to the electrode, which reduces the defective state and the amount of charged impurities or molecules between the light-absorption layer and the electrode, increasing transport efficiency and enhancing the photoresponsivity [26]. In addition, it is evident that this is due to the variation in the intensity of the incident light photons and the increase in the drift rate of the carriers, resulting in a device with excellent performance at low power, providing a method for future studies of low power sensors. It is well known that due to the short wavelength of light having a larger energy level, the energy of light exceeding the bandgap can excite more electrons from the valence band to the conduction band, and the probability of leap increases, which greatly enhances the photocurrent of HDA-BiI_5_ devices.

**Figure 3:**
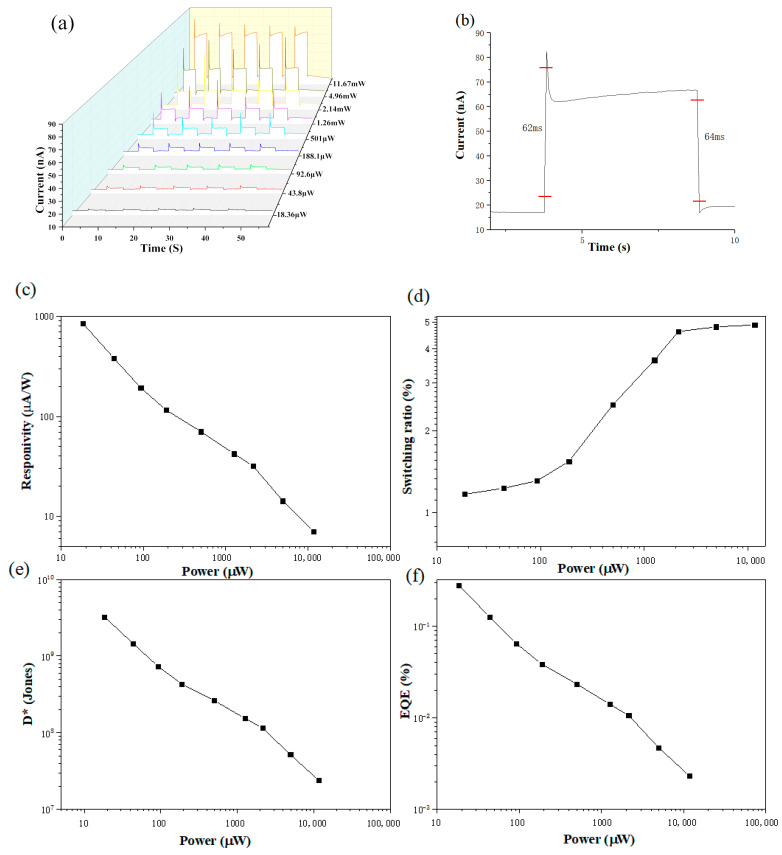
The time-response and photoresponse characteristics of HAD-BiI_5_-based photodetectors under a zero-bias 532 nm laser: (**a**) photocurrent versus time plots of device; (**b**) response time of HDA-BiI_5_-based photodetector (532 nm, 11.67 mW); (**c**) photoresponsivity; (**d**) switching ratio; (**e**) specific detectivity; (**f**) external quantum efficiency.

We tested the photoelectronic performance using a 532 nm wavelength laser. Reproducibility and photoresponse speed are among the most important parameters of photodetectors, so preparing detectors with high performance is still a challenge. As shown in Figure 3a, we also perform five light-switching operations, and the photocurrent does not change significantly, indicating that the device also has good photocurrent stability at 532 nm. For photodetector applications, fast response and decay speed are usually required characteristics. Figure 3b shows that we take the time from 10% to 90% of the response as the rise time and decay time. It can be seen that the device rise time is about 62 ms and the decay time is about 64 ms. In addition, the photoresponsivity can reach up to 8.5 × 10^−4^ A/W. According to previous reports, this value is outstanding among the HAD-BiI5 photodetectors. This indicates that the device can respond and recover quickly even under the irradiation of light with larger wavelengths, but the photocurrent is not as high as that under 375 nm, mainly because the low energy of light leads to fewer excited electron-hole pairs, which contribute little to the increase of the photocurrent.

As can be seen in Figure 3, all the characteristic indicators have decreased compared to 375 nm, but the general trend is the same as in Figure 2. The trends of specific detectivity and quantum efficiency are similar to that of the photoresponsivity curve. However, the performance under 532 nm laser irradiation is reduced compared to that under 375 nm laser irradiation. With the increase of optical power, the photoresponsivity is gradually decreasing, but it can still reach 8.5 × 10^−4^ A/W. At this time, the photoresponsivity is still much higher than that of the same type of photodetector [12]. It shows that the device has excellent performance, and also has the characteristics of multi-band detection and fast response, which provide a reference for the preparation of organic–inorganic photodetectors in the future.

## 4. Conclusions

The photodetector was prepared using the organic–inorganic hybrid semiconductor material hexane-1,6-diammonium pentaiodobismuth (HDA-BiI_5_), and the photodetector was tested under a single light source at 532 nm and 375 nm, respectively, to study the photoresponse and time-response characteristics of the HDA-BiI_5_-based photodetector. We also found that this device has high photoresponsivity and a fast response time. In addition, the effect on the device performance of switching the light on and off at different power levels is studied, with the device being found to have good stability and repeatability. The results of the above study help to enhance the scope of the application of organic–inorganic materials in photodetectors.

## Figures and Tables

**Table 1:** Performance comparison of HDA-BiI_5_-based photodetector with other similarly structured materials devices.

Photodetector	ETL (Yes/No)	Polarization (Yes/No)	Light (nm)	Dark- Current (pA)	Responsivity (A/W)	Detectivity [Jones]	Rise and Decay Time (s)	Ref.
BaTiO_3_	No	No	365	-	3.48 × 10^−9^	2.06 × 10^4^	-	[7]
PLZT8	No	No	405	>22	4.48 × 10^−7^	7.15 × 10^7^	-	[20]
BaTiO_3_	No	No	405	>100	<3.5 × 10^−7^	<3.5 × 10^5^	0.4/1.6	[21]
BiFeO_3_	No	Yes	450	>100	~10^−7^	~10^8^	0.5/0.8	[22]
PLZT	No	Yes	375	3	<5 × 10^−5^	<9.52 × 10^8^	>0.42/0.46	[8]
	No	Yes	532		<2.5 × 10^−5^	<9.52 × 10^8^	0.42/0.46	
	No	Yes	375	2 2	<1 × 10^−4^	<3.69 × 10^9^	0.34/0.36	
	No	Yes	532		<2.5 × 10^−5^	<3.69 × 10^9^	>0.34/0.36	
BaTiO_3_	No	No	405	-	∼10^−7^	10^5^	0.6/0.5	[23]
BaTiO_3_	No	No	365	-	∼10^−7^	-	0.56/13.44	[24]
TiO_2_:P3HT	Yes (TiO_2_)	No	375	>10^3^	<5 × 10^−4^	<10^−8^	>0.52/0.87	[25]
	Yes (TiO_2_)	No	532		<4.5 × 10^−4^	<10^−8^	>0.52/0.87	
HDA-BiI_5_	Yes (TiO_2_)	Yes	Sunlight illumination	-	-	-	-	[11]
HDA-BiI_5_	Yes (TiO_2_)	No	Sunlight illumination	-	-	-	-	[10]
HDA-BiI_5_	No	No	375	16	5.37 × 10^−^^4^	5.9 × 10^10^	0.061/0.062	[12]
	No	No	532		1.28 × 10^−4^	1.4 × 10^10^	0.062/0.063	
HDA-BiI_5_	Yes (SnO_2_)	No	375	20	1.45 × 10^−3^	5.0 × 10^9^	0.063/0.062	This work
	Yes (SnO_2_)	No	532		8.5 × 10^−^^4^	3.2 × 10^9^	0.062/0.064	

## Data Availability

Not applicable.
